# Combination of the Probiotics *Lacticaseibacillus rhamnosus* GG and *Bifidobacterium animalis* subsp. *lactis*, BB-12 Has Limited Effect on Biomarkers of Immunity and Inflammation in Older People Resident in Care Homes: Results From the Probiotics to Reduce Infections iN CarE home reSidentS Randomized, Controlled Trial

**DOI:** 10.3389/fimmu.2021.643321

**Published:** 2021-03-04

**Authors:** Vivian M. Castro-Herrera, Helena L. Fisk, Mandy Wootton, Mark Lown, Eleri Owen-Jones, Mandy Lau, Rachel Lowe, Kerenza Hood, David Gillespie, F. D. Richard Hobbs, Paul Little, Christopher C. Butler, Elizabeth A. Miles, Philip C. Calder

**Affiliations:** ^1^School of Human Development and Health, Faculty of Medicine, University of Southampton, Southampton, United Kingdom; ^2^Specialist Antimicrobial Chemotherapy Unit, Public Health Wales, University Hospital of Wales, Cardiff, United Kingdom; ^3^School of Primary Care and Population Sciences, Faculty of Medicine, University of Southampton, Southampton, United Kingdom; ^4^Centre for Trials Research, Cardiff University, Cardiff, United Kingdom; ^5^Nuffield Department of Primary Care Health Sciences, University of Oxford, Oxford, United Kingdom; ^6^National Institute for Health Research (NIHR) Southampton Biomedical Research Centre, University Hospital Southampton National Health Service (NHS) Foundation Trust and University of Southampton, Southampton, United Kingdom

**Keywords:** care home residents, aging, probiotic, immunity, inflammation, immunosenescence, inflammageing

## Abstract

Aging is associated with a decline in many components of the immune system (immunosenescence). Probiotics may improve the immune response in older people. The objective was to determine the effect of the combination of two probiotic organisms [*Lacticaseibacillus* (previously known as *Lactobacillus*) *rhamnosus* GG (LGG) and *Bifidobacterium animalis* subsp. *lactis*, BB-12 (BB-12)] on a range of immune biomarkers measured in the blood of older people resident in care homes in the UK. In a randomized controlled trial, older people [aged 67–97 (mean 86) years] resident in care homes received the combination of LGG+BB-12 (1.3–1.6 × 10^9^ CFU per day) or placebo for up to 12 months. Full blood count, blood immune cell phenotypes, plasma immune mediator concentrations, phagocytosis, and blood culture responses to immune stimulation were all measured. Response to seasonal influenza vaccination was measured in a subset of participants. Paired samples (i.e., before and after intervention) were available for 30 participants per group. LGG and BB-12 were more likely to be present in feces in the probiotic group and were present at higher numbers. There was no significant effect of the probiotics on components of the full blood count, blood immune cell phenotypes, plasma immune mediator concentrations, phagocytosis by neutrophils and monocytes, and blood culture responses to immune stimulation. There was an indication that the probiotics improved the response to seasonal influenza vaccination with significantly (*p* = 0.04) higher seroconversion to the A/Michigan/2015 vaccine strain in the probiotic group than in the placebo group (47 vs. 15%).

## Introduction

Aging is associated with changes in immunity, collectively termed immunosenescence (1–3) and inflammageing (4, 5). Immunosenescence describes impairments in neutrophil, antigen presenting cell, T cell and B cell function (6–8) which increase susceptibility to infection (9, 10) and diminish responses to vaccination (11, 12). Inflammageing describes an elevated state of chronic low-grade inflammation which is considered to increase risk of non-communicable diseases (13, 14). Together these age-related changes in immunity contribute to poor quality of life, increased illness and mortality. Increased infection in older people results in increased use of antibiotics (15), contributing to emergence of antibiotic resistant bacterial strains (16–18). Therefore, strategies to slow or reverse immunosenescence and inflammageing could play an important role in improving health and well-being in older people and result in reduced health and social care costs.

The gut microbiota has also been described to be altered in older people (19, 20), with age-related changes being accelerated by residence in a care home (21). These changes might be related to immune decline and inflammageing, since the gut microbiota plays a role in regulating the host immune and inflammatory responses (22). Probiotics can be used to beneficially modify the gut microbiota (23) and this in turn could improve host immunity and dampen low-grade inflammation (24). The most effective probiotics, and therefore the most widely studied, seem to be lactobacilli and bifidobacteria (25), including *Lacticaseibacillus* (previously known as *Lactobacillus*) *rhamnosus* GG (LGG) and *Bifidobacterium animalis* subsp. *lactis*, BB-12 (BB-12). These organisms have been shown to improve constipation (26) and reduce diarrheal episodes caused by pathogenic organisms in older people (27). LGG and BB-12 have also been reported to reduce inflammation in the gut in older people (27). It has also been shown in older institutionalized individuals that inflammatory responses measured through levels of tumor necrosis factor (TNF)-α are influenced by bifidobacteria over 6 months of consumption (28). Despite these findings, there is a lack of studies of LGG and BB-12 on immunity and inflammation in older people resident in care homes.

The Probiotics to Reduce Infections iN CarE home reSidentS (PRINCESS) trial is a randomized, placebo-controlled trial of the combination of LGG and BB-12 in older people resident in care homes with the primary outcome being antibiotic use (29). The primary and a number of secondary outcomes of the PRINCESS trial are reported elsewhere (30). Importantly, the likelihood of colonization with both LGG and BB-12 and the number of both LGG and BB-12 present in feces were significantly higher in the group receiving LGG plus BB-12 compared to the placebo group (30). Here we report a range of immune and inflammatory markers for participants in the PRINCESS trial; we assessed static measures in blood [full blood count, immune phenotypes, plasma immune mediator and C-reactive protein (CRP) concentrations] as well as blood immune cell responses after *ex vivo* challenge (phagocytosis, blood culture responses to immune stimulation) and included components of both innate and acquired immunity. We also assessed response to seasonal influenza vaccination in a subset of participants.

## Materials and Methods

### Participants

**Figure 1:**
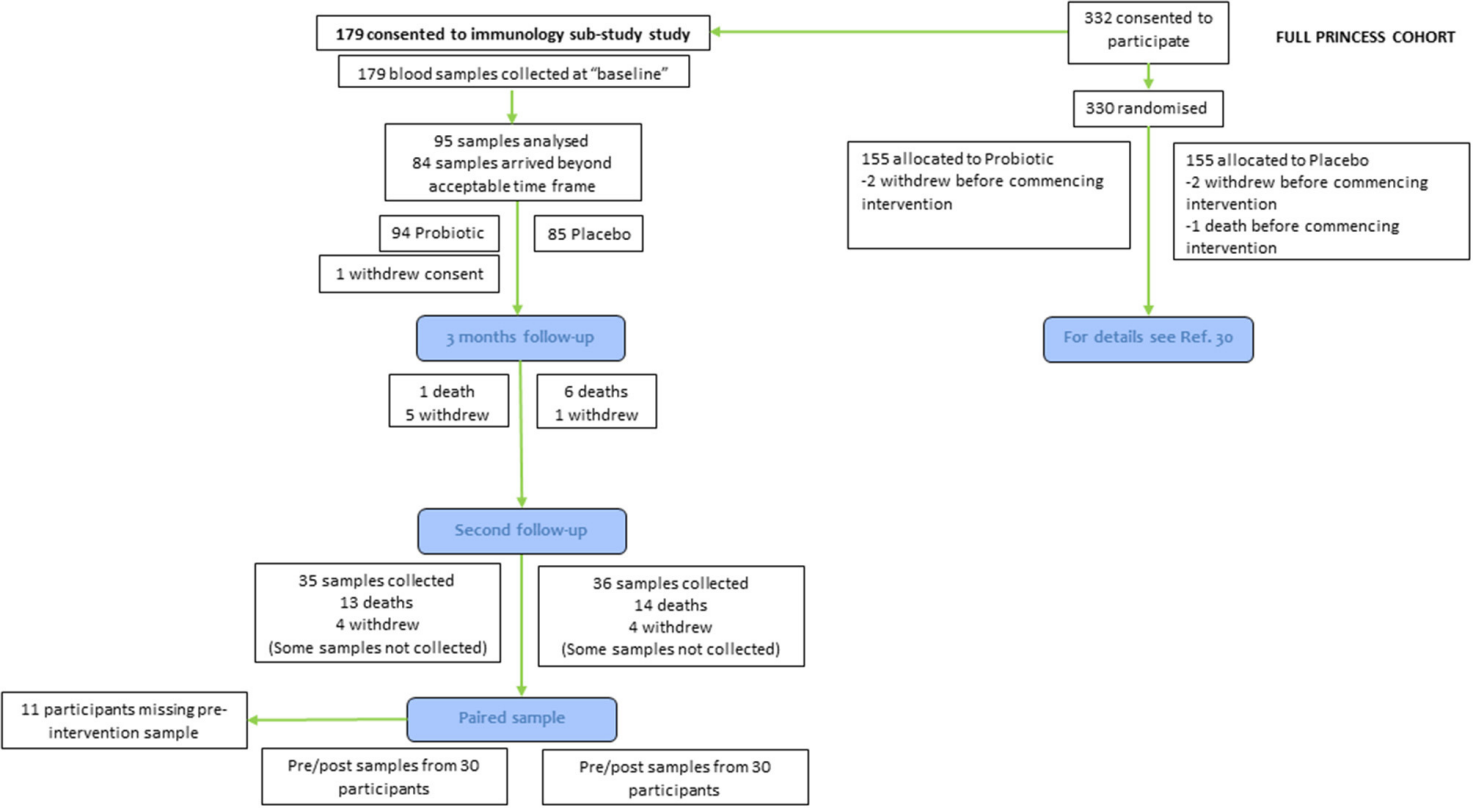
Flow of participants through the study.

The PRINCESS trial was a two-arm double-blind individually-randomized placebo controlled trial, involving three academic centers in the UK (Universities of Cardiff, Oxford and Southampton). The full protocol (29) and the main outcomes (30) of the PRINCESS trial have been published. The PRINCESS trial was approved by the Wales REC 3 (15/WA/0306) and is registered as ISRCTN16392920. Care home residents were eligible for participation if they were aged 65 yr or older and willing and able to give informed consent for participation; if they lacked capacity, a consultee could complete a consultee declaration for participation on their behalf. Exclusions were immunocompromise (ongoing immune-suppressants; long-term, high-dose, oral, intramuscular or intravenous steroids), lactose intolerance, taking ongoing regular probiotics, or temporary residence in the care home. Care homes were residential, nursing or mixed. Here we report immune and inflammatory outcomes in a subset of participants recruited into the PRINCESS trial (60 out of 310 participants) (Figure 1). Data were not available for all participants because (a) some participants did not consent to take part in the immune sub-study of PRINCESS; or (b) insufficient blood was collected to measure some or any of the immune parameters; or (c) the blood arrived at the University of Southampton, where immune measurements were made, outside of a time window pre-determined based upon an earlier study (31).

### Interventions

Participants were randomized using an online process in a 1:1 ratio using minimization to balance groups by care home and resident sex to daily oral combination of LGG and BB-12 (total cell count 1.3 × 10^9^ to 1.6 × 10^9^) or a matched placebo (containing maltodextrin, microcrystalline cellulose, magnesium stearate, and silicon dioxide) in a capsule (both supplied by Chr. Hansen A/S, Hørsholm, Denmark); these were not administered while participants were away from care homes such as when hospitalized. A total of 310 residents (155 in each group) were randomized from 23 care homes in the UK between December 2016 and May 2018. The duration of intervention was initially set at 365 days. However, due to slower than anticipated recruitment, follow-up was truncated for 106 care home residents; for these participants the second follow-up occurred between 6 and 11 months post-randomization. The end of study timepoint for all participants is referred to as the second follow-up (Figure 1).

### Assessment of Frailty

Frailty index was determined according to the scale described elsewhere (32). The scale has nine categories defined as: 1 = Very fit for their age (active, energetic and motivated); 2 = Well (absent symptomatology of disease but less active); 3 = Managing well (medical problems under control but not regularly active); 4 = Vulnerable (symptoms that limit activities but not decedent on others); 5 = Mildly frail (impairment of daily activities); 6 = Moderately frail (progressive impairment and declined activities); 7 = Severely frail (completely dependent cognitively or physically but not terminally ill); 8 = Very severely frail (completely dependent and approaching the end of life); 9 = Terminally ill (life expectancy < 6 mo).

### Assessment of Fecal LGG and BB-12

Fecal samples were collected at study entry, after 3 months of intervention and again at the second follow-up. A small (ball 5 mm in diameter) sample of feces was used to inoculate 3 mL saline and then 50 μl of this inoculate was spiral plated (Dan Whitley Ltd., UK) onto selective agar to isolate relevant bacteria. Lactobacillus Selective Agar (LBS) and Bifidobacterium Agar (BA) were used for probiotic detection (Becton Dickinson, UK). LBS plates were incubated at 35 ± 1°C in a CO_2_ atmosphere for 24–72 h and BA plates were incubated anaerobically at 35 ± 1°C for 24–72 h. A quantitative count of bacteria [colony forming units per ml of the 3 ml saline suspension (CFU/ml)] of bacteria was performed using the Don Whitley Ltd. counting calculator for the morphologically-defined isolates on the selective media. Specific organisms were identified by Matrix-Assisted Laser Desorption Ionization-Time of Flight (MALDI-ToF) mass spectrometry using the MALDI Biotyper® technology (Bruker, UK). MALDI-TOF mass spectrometry determines the unique proteomic fingerprint of an organism, and matches characteristic patterns with an extensive reference library (Bruker, UK) to determine the organism's identity.

### Assessment of Immune and Inflammatory Biomarkers

Blood was collected into EDTA or heparin at the care homes at study entry and at the end of the intervention period (i.e., at the second follow-up) and was posted to the University of Southampton. Whole blood was used to determine full blood count, for immune phenotyping by flow cytometry, for assessment of neutrophil and monocyte phagocytosis, and for cultures to determine production of immune mediators after incubation with different immune stimulants. Plasma was prepared for measurement of CRP and immune mediator concentrations. These measurements were all made as described elsewhere (31). In addition, 39 participants (*n* = 19 probiotic group and *n* = 20 in the placebo group) received the 2017/2018 quadrivalent seasonal influenza vaccine. This vaccine contained the A/Brisbane/60/2008-like virus, A/Michigan/45/2015 (H1N1)pdm09-like virus, A/Hong Kong/4801/2014 (H3N2)-like virus, and B/Phuket/3073/2013-like virus. Participants were already consuming the probiotics or placebo for a median of 5.8 months (SD 2.3 months) at the time of vaccination. A blood sample was collected 5–15 days prior to vaccination and then 31–39 days after vaccination. Antibody titres against each viral strain were measured in serum samples at the National Infection Service laboratory, Public Health England, Colindale, London, UK. Viral titres, seroconversion (at least a 4-fold increase in antibody titer) and seroprotection (an antibody titer of ≥40 HI units) are all presented.

## Statistical Analysis

The sample size of the PRINCESS trial was based on a 10% relative reduction in cumulative systemic antibiotic administration days, assumed at 17.4 days per resident-year in the placebo group and a reduction in the probiotic group to 15.6 days per resident-year; such a 10% reduction was considered feasible and clinically important. An estimated 330 participants would provide 90% power at the 5% level to demonstrate this effect. No formal power calculation was performed for the outcomes reported here. Data collation and analysis were performed in SPSS version 22 (IBM Corp. Released 2013. IBM SPSS Version 22.0. Armonk, New York), Excel and GraphPad Prism 8.2.1. Normality of data was assessed by visual inspection of histogram distributions and by using the Shapiro Wilk and Kolmogorov-Smirnov tests. Most immune biomarker data were not normally distributed and so were log transformed to fit a regression model. Analyses of immune biomarkers were adjusted by allocation (trial arm - either placebo or probiotic), sex and baseline measurement through analysis of covariance (ANCOVA) using post-intervention outcome as the dependent variable. Variables whose characteristics did not fit ANCOVA assumptions were analyzed through the Mann-Whitney test. Fecal LGG and BB-12 and vaccine titres were analyzed using the Mann-Whitney test and the Fisher's exact test. In all cases, statistical significance was inferred by a value for *p* < 0.05. We did not correct for multiple comparisons.

## Results

Participants included in this immunology sub-study (*n* = 60 who had usable paired samples available from before and after the intervention) had similar characteristics to those in the main PRINCESS cohort (Table 1). Participants in the sub-study were aged 67–97 (mean 86; SD 6.6) yr and 46% were male. Of the 60 participants reported here, 58 consumed placebo or probiotics for more than 6 months, 52 for more than 7 months, 48 for more than 8 months, 49 for more than 9 months, 44 for more than 10 months, 38 for more than 11 months and 23 for 12 to 13.5 months. Mean duration of consumption of placebo or probiotics did not differ and was not different from the duration seen in the main cohort (Table 1). Mean CRP concentration at baseline was 6.3 mg/l; the distribution of CRP concentrations was skewed and the median concentration was 3 mg/l.

**Table 1:** General characteristics of the participants in the PRINCESS trial (main cohort) and in the immunology sub-study.

	**Immunology sub-study**	**Main cohort**
	**(*n* = 60)**	**(*n* = 310)**
**Variable**	**Mean ± SD**	**Mean ± SD**
Age (yr)	86.2 ± 6.6	85.3 ± 7.4
Duration of care home residence (y)	1.4 ± 1.6	1.7 ± 2.4
Height (cm)	1.7 ± 0.7	1.6 ± 0.1
Weight at baseline (kg)	69.8 ± 16.7	64.3 ± 15.9
Middle upper arm circumference (cm)	27.7 + 3.4	27.2 ± 4.3
Length of consumption probiotics (d)	263 ± 102	239 ± 107
Length of consumption placebo (d)	253 ± 109	213 ± 112
**Frailty index [*****n*** **(%)]:**		
1	1 (1.7)	4 (1.3)
2	1 (1.7)	8 (2.6)
3	6 (10.0)	19 (6.1)
4	4 (6.7)	11 (3.5)
5	8 (13.3)	20 (6.5)
6	20 (33.3)	84 (27.1)
7	20 (33.3)	158 (51.0)
8	0 (0)	6 (1.9)
9	0 (0)	0 (0)

Data on fecal LGG and BB-12 are shown in Table 2. About 27% of participants were colonized with LGG at study entry (i.e., they had LGG in their feces); this increased to 79% in the probiotic group at 3 months and 72% at the second follow-up. The number of fecal LGG was low in the placebo group throughout the study but was higher at the second follow-up in the probiotics group than in the placebo group (Table 2). Very few participants (2.5%) were colonized with BB-12 at study entry (i.e., had BB-12 in their feces). By 3 months over 55% of participants in the probiotics group were colonized with BB-12. At this timepoint, no participants in the placebo group were colonized with BB-12. The number of fecal BB-12 was low in the placebo group throughout the study, but was higher in the probiotics group than in the placebo group at both 3 months and the second follow-up (Table 2).

**Table 2:** Fecal *Lacticaseibacillus rhamnosus* GG and *Bifidobacterium animalis* subsp. *lactis*, BB-12 in participants in the placebo and probiotic groups.

	**Placebo**	**Probiotic**	***P***
***Lacticaseibacillus rhamnosus*** **GG**			
Present at study entry (%)	26.3	27.8	1.000^b^
Present at 3 months (%)	37.5	78.9	0.018^b^
Present at second follow-up (%)	37.5	72.2	0.082^b^
Median number (CFU/ml) at study entry (IQR)	0 (0–10)	0 (0–1,560)	0.773^a^
Median number (CFU/ml) at 3 months (IQR)	0 (0–10,800)	1200 (180–36,500)	0.082^a^
Median number (CFU/ml) at second follow-up (IQR)	0 (0–1,350)	29,000 (0–77,100)	0.046^a^
***Bifidobacterium animalis*** **subsp**. ***lactis**,* **BB-12**			
Present at study entry (%)	0	5.3	1.000^b^
Present at 3 months (%)	0	57.9	<0.001^b^
Present at second follow-up (%)	12.5	55.6	0.013^b^
Median number (CFU/ml) at study entry (IQR)	0 (0-0)	0 (0-0)	0.795^a^
Median number (CFU/ml) at 3 months (IQR)	0 (0-0)	15000 (0-2,800,000)	0.003^a^
Median number (CFU/ml) at second follow-up (IQR)	0 (0-0)	2300 (0-180,000)	0.053^a^

a *Mann-Whitney Test*.

b *Fisher's Exact Test*.

*IQR, Interquartile Range*.

This probiotic intervention did not influence parameters included in the full blood count (numbers of leukocytes, neutrophils, basophils, eosinophils, lymphocytes, monocytes, platelets); blood immune phenotypes (numbers of T lymphocytes, helper T lymphocytes, cytotoxic T lymphocytes, activated cytotoxic T lymphocytes, regulatory T lymphocytes, natural killer cells, B lymphocytes, activated B lymphocytes, monocytes, activated monocytes and ratio of CD4^+^ (helper T lymphocytes) to CD8^+^ (cytotoxic T lymphocytes) cells); phagocytosis of *Escherichia coli* by neutrophils and monocytes; plasma concentrations of CRP and 12 different immune mediators [TNF-α, interleukin (IL)-1 receptor antagonist, IL-6, IL-10, IL-12p70, IL-17A, TNF receptor 2, soluble intercellular adhesion molecule 1, soluble E-selectin, soluble vascular cell adhesion molecule 1, monocyte chemoattractant protein 1, interferon gamma-induced protein 10]; or the production of immune mediators by whole blood cultures stimulated with bacterial lipopolysaccharide, peptidoglycan or phytohemagglutinin (Supplementary Tables 1–7). In the regression model neither treatment allocation nor sex was a significant predictor of any of the outcomes at the end of intervention, but baseline value was a significant predictor of end of intervention value in most cases (Supplementary Tables 1–7).

A subset of participants (*n* = 39) received the seasonal influenza vaccination. Data on antibody titres, seroprotection and seroconversion in these participants are shown in Table 3. A high proportion of participants were seroprotected prior to vaccination: 41, 77, 95, and 85% of participants were seroprotected against A/Michigan/2015, A/Hong Kong/2014, B/Brisbane/2008 and B/Phuket/2013, respectively (Table 3). Post-vaccination antibody titres to any of the vaccine strains and seroprotection did not differ between groups (Table 3). The percentage of participants who were seroprotected after but not before vaccination was numerically higher for all four vaccine strains in the probiotic group, but this was not significant between groups (Table 3). Seroconversion to the A/Michigan/2015 vaccine strain was significantly higher (*p* = 0.04) in the probiotic group than in the placebo group (47 vs. 15%; Table 3).

**Table 3:** Anti-seasonal influenza antibody titres, seroprotection and seroconversion in participants in the placebo and probiotic groups.

	**Placebo** ** (*n* = 20)**	**Probiotic** ** (*n* = 19)**	***P***
**A/Michigan/2015**			
Antibody titer prior to vaccination (HI) [Median (IQR)]	25 (10–120)	10 (10–80)	0.296^a^
Antibody titer after vaccination (HI) [Median (IQR)]	140 (10–300)	80 (20–160)	0.901^a^
Median (IQR) fold increase	1.7 (1–3)	2.0 (1–8)	0.184^a^
Seroprotection prior to vaccination (%)	50	32	0.333^b^
Seroprotection after vaccination (%)	65	74	0.731^b^
Seroprotected after but not prior to vaccination (%)	15	42	0.189^b^
Seroconversion (%)	15	47	0.040^b^
**A/Hong Kong/2014**			
Antibody titer prior to vaccination (HI) [Median (IQR)]	160 (25–320)	160 (60–320)	0.945^a^
Antibody titer after vaccination (HI) [Median (IQR)]	400 (80–880)	320 (80–960)	0.813^a^
Median (IQR) fold increase	1.8 (1–4)	2.0 (1–4)	0.879^a^
Seroprotection prior to vaccination (%)	75	79	1.00^b^
Seroprotection after vaccination (%)	85	95	0.605^b^
Seroprotected after but not prior to vaccination (%)	10	16	0.738^b^
Seroconversion (%)	25	26	1.0^b^
**B/Brisbane/2008**			
Antibody titer prior to vaccination (HI) [Median (IQR)]	320 (100–640)	320 (80–480)	0.588^a^
Antibody titer after vaccination (HI) [Median (IQR)]	320 (160–1,280)	480 (320–960)	0.749^a^
Median (IQR) fold increase	1.7 (1–2)	2.0 (1–4)	0.270^a^
Seroprotection prior to vaccination (%)	100	90	0.231^b^
Seroprotection after vaccination (%)	100	95	0.487^b^
Seroprotected after but not prior to vaccination (%)	0	5	0.231^b^
Seroconversion (%)	20	26	0.716^b^
**B/Phuket/2013**			
Antibody titer prior to vaccination (HI) [Median (IQR)]	160 (80–320)	80 (60–320)	0.235^a^
Antibody titer after vaccination (HI) [Median (IQR)]	320 (160–600)	320 (150–480)	0.380^a^
Median (IQR) fold increase	2.0 (1–4)	2.0 (1–4)	0.879^a^
Seroprotection prior to vaccination (%)	90	79	0.407^b^
Seroprotection after vaccination (%)	100	95	0.487^b^
Seroprotected after but not prior to vaccination (%)	10	16	0.230^b^
Seroconversion (%)	30	37	0.741^b^

a *Mann-Whitney test*.

b *Fisher's Exact Test*.

*IQR, Interquartile Range*.

The participant population included individuals at various stages of frailty (Table 1). Therefore, as an *a posteriori* investigation, we examined whether the response to vaccination among participants differed according to whether they were less frail [category 5 (mildly frail) or 6 (moderately frail) *n* = 25] or more frail [category 7 (severely frail) *n* = 19], according to the frailty index described in Section Assessment of Frailty. Less frail individuals appeared more likely to be seroprotected prior to vaccination, although the differences between frailty groups were not significant (Table 4). Post-vaccination antibody titres to any of the vaccine strains and seroprotection did not differ between frailty groups (Table 4). The percentage of participants who were seroprotected after but not before vaccination was numerically higher for all four vaccine strains in the less frail group, but was not significantly different between groups (Table 4). The percentage of participants achieving seroconversion for all four vaccine strains was numerically higher in the less frail group, but was not statistically significantly different between groups (Table 4).

**Table 4:** Anti-seasonal influenza antibody titres, seroprotection and seroconversion in participants according to frailty.

	**Less frail** ** (*n* = 25)**	**More frail** ** (*n* = 14)**	***P***
**A/Michigan/2015**			
Antibody titer prior to vaccination (HI) [Median (IQR)]	10 (10–80)	10 (10–90)	0.919^a^
Antibody titer after vaccination (HI) [Median (IQR)]	80 (10–200)	100 (10–320)	0.919^a^
Median (IQR) fold increase	2 (1–4)	2 (1–3)	0.828^a^
Seroprotection prior to vaccination (%)	25.6	15.4	0.563^b^
Seroprotection after vaccination (%)	46.2	23.1	0.440^b^
Seroprotected after but not prior to vaccination (%)	20.6	7.7	0.838^b^
Seroconversion (%)	23	8	0.477^b^
**A/Hong Kong/2014**			
Antibody titer prior to vaccination (HI) [Median (IQR)]	120 (20–320)	200 (80–400)	0.331^a^
Antibody titer after vaccination (HI) [Median (IQR)]	320 (80–800)	400 (260–960)	0.426^a^
Median (IQR) fold increase	2 (1–4)	2 (1–2)	0.784^a^
Seroprotection prior to vaccination (%)	46.2	30.8	0.288^b^
Seroprotection after vaccination (%)	56.4	33.3	0.545^b^
Seroprotected after but not prior to vaccination (%)	10.2	2.5	0.736^b^
Seroconversion (%)	21	5	0.721^b^
**B/Brisbane/2008**			
Antibody titer prior to vaccination (HI) [Median (IQR)]	320 (120–560)	240 (80–400)	0.460^a^
Antibody titer after vaccination (HI) [Median (IQR)]	640 (320–1,280)	240 (160–640)	0.020^a^
Median (IQR) fold increase	2 (1–4)	1 (1–2)	0.062^a^
Seroprotection prior to vaccination (%)	61.5	33.3	0.595^b^
Seroprotection after vaccination (%)	64.1	33.3	0.359^b^
Seroprotected after but not prior to vaccination (%)	2.6	0	0.595^b^
Seroconversion (%)	23	0	0.119^b^
**B/Phuket/2013**			
Antibody titer prior to vaccination (HI) [Median (IQR)]	80 (80–320)	160 (80–320)	0.443^a^
Antibody titer after vaccination (HI) [Median (IQR)]	320 (160–480)	160 (150–520)	0.515^a^
Median (IQR) fold increase	2 (2–4)	2 (1–3)	0.195^a^
Seroprotection prior to vaccination (%)	53.8	30.8	0.635^b^
Seroprotection after vaccination (%)	61.5	35.9	0.641^b^
Seroprotected after but not prior to vaccination (%)	7.7	5.1	1.000^b^
Seroconversion (%)	26	8	0.304^b^

a *Mann-Whitney test*.

b *Fisher's Exact Test*.

*IQR, Interquartile Range*.

## Discussion

Older people can exhibit immune decline, termed immunosenescence, low-grade inflammation, termed inflammageing, and an altered gut microbiota. These may be inter-related. Probiotic bacteria colonize the intestine and interact with the gut-associated lymphoid tissue (33–35). Some studies report that probiotic bacteria enhance markers of immunity in older people (35–37). The current study investigated the combination of two probiotic strains, LGG and BB-12, given daily for at least 6 months, in older people resident in care homes in the UK. The participants had an average age of 86 yr, were fairly frail and had a mean plasma CRP concentration of 6.3 mg/l (median 3 mg/l), confirming low grade inflammation amongst many of them. Gut colonization by both LGG and BB-12 was demonstrated by positive cultures from fecal samples. This is important because such colonization is believed to be the basis of the ability of probiotics to modify host immune response (23). Nevertheless, there was no significant effect of the probiotics on blood immune cell numbers or subtypes, circulating markers of immunity and inflammation, cellular responses measured through phagocytic responses and secretion of immune mediators following exposure to three different immune stimulants. These observations suggest that gut colonization by LGG and BB-12 is not associated with generalized improvements in immune function in older people resident in care homes. Consistent with this finding, the primary outcome of the PRINCESS trial, antibiotic use, was not reduced by probiotics (30). We also studied response to seasonal influenza vaccination in a sub-set of participants. There were indications that responses to vaccination (seroprotection and seroconversion) were numerically greater in the probiotic group than in the placebo group, although this was significant only for seroconversion to the A/Michigan/2015 strain. Lack of significance of the other findings may be due to the small sample size. It was observed that a number of participants were seroprotected prior to vaccination. In the current study the 2017/2018 quadrivalent vaccine was used. Three of the viral strains (A/Hong Kong/4801/2014 (H3N2)-like, B/Brisbane/60/2008-like and B/Phuket/3073/2013-like) had also been included in the 2016/2017 vaccine. Thus, participants may have been exposed previously to these strains through vaccination in the previous year. We have no access to the vaccination records of the participants to confirm this, but it seems likely because of the participants age. Nevertheless, a number of participants did respond to the vaccination, as detected through increased antibody titer and seroconversion. The A/Michigan/45/2015 (H1N1)pdm 09-like strain had not been used in a previous vaccine, yet about 40% of participants were already seroprotected prior to vaccination. This might suggest exposure to the influenza virus itself among these participants. We also identified that responses to vaccination were numerically greater in those who were less frail, which is consistent with frailty and impaired immunity being related (38).

LGG and BB12 have been reported to modulate immune responses in humans (28, 39–41), in animals (42, 43) and *in vitro* (44, 45). However, this was not observed in the current study, although the numerically higher vaccination responses in the probiotic group may suggest an effect that was not sufficiently strong to be detected as significant because of the limitation on sample size. It is possible that the participants in the current study were too frail for their immune system to respond to the probiotic intervention. A previous study reported that the consumption of *B. lactis HN019* by older volunteers (aged 63–84 yr) for 9 weeks increased the number of blood helper T cells (CD4^+^) and activated (CD25^+^) T cells as well as NK cells (36). Furthermore, another study reported that healthy middle aged and older individuals (41–81 yr; median age: 60 yr) exhibited increased NK cell activity following an intervention with *B. lactis* HN019 when compared with the placebo group (low-fat milk as carrier alone) (46). In the current study circulating T cell and NK cell numbers were not altered by the probiotics. It is likely that participants in both these previous studies were less frail than those in the current study and many were younger.

Antibody production is a surrogate indicator of B cell function and it has been described that B cell numbers do not change with age progression, but rather they suffer an impairment in their ability to produce antibodies (47). It has been shown that reduced expression of genes encoding for immunoglobulin class switch recombination as well as altered mechanisms of somatic hypermutation (involved in antibody production by B cells) have a detrimental impact on humoral immune responses (48) with subsequent reduced responses with new antigenic challenges and thus poorer responses to vaccination and to new infections. In the current study the probiotics LGG plus BB-12 did not alter circulating B cell numbers, but may have had a modest effect on B cell function as indicated by the numerically higher vaccination responses in the probiotic group.

The findings of the current study may be compared with those of previous studies investigating the impact of lactobacilli on vaccination responses in older people. Boge et al. (37) conducted a pilot study in 68 healthy older adults (mean age ~84 yr) in nursing homes and then conducted a confirmatory study in 222 older adults (mean age ~84 yr) in the same setting. They found that compared with placebo, daily *L. casei* DN-114 001 (also known as *L. paracasei* subsp. *paracasei*) for 7 weeks in the pilot and then 13 weeks in the confirmatory study improved the response to influenza vaccination (37). A study in 15 healthy adults aged 65–85 y in nursing homes given *L. plantarum* daily for 3 months found increased influenza-virus specific IgA and IgG antibodies following vaccination (35). It is possible that the effects of probiotics on immunity are strain specific (49) or that frailty limits the effectiveness of probiotics (50).

The current study has several strengths. There were few restrictions on participant inclusion, so long as they were care home residents. The period of intervention was of significant duration (>6 months for 58 out of 60 participants) and gut colonization with LGG and BB-12 was confirmed. A broad range of immune and inflammatory outcomes were measured, representing several different components of the immune system; these included static measures in blood (full blood count, immune phenotypes, plasma mediators) as well as cell responses after challenge (phagocytosis, blood culture responses to LPS, PGN and PHA) and components of innate (phagocytosis, blood culture responses to LPS and PGN) and acquired immunity (blood culture responses to PHA). Finally, response to vaccination, considered the most robust marker of immune function (51, 52), was assessed. However, the study also has some limitations. Firstly, not all participants in the full PRINCESS trial agreed to participate in the immunology sub-study. Secondly, not all samples were available for those who did participate; this is mainly because some blood samples did not arrive at the laboratory within a predetermined time to assure the reliability of the data (31). This meant that paired samples (before and after intervention) were available for 60 participants (30 per group). Thirdly, only a small number of participants became involved in the seasonal influenza vaccination component of the study. Fourthly, because of a time limitation on completing the study, some participants were involved for less the 12 months. Fifthly, as all outcomes reported here are pre-defined secondary outcomes of the PRINCESS trial, no power calculation was done. Finally, the statistical analysis is not corrected for multiple comparisons, so the few significant effects could have arisen by chance.

It has proven to be difficult to consistently demonstrate effects of probiotics (and prebiotics) on markers of immune function in humans (53, 54). One reason for this may be the large between-individual variations that exist in most immune markers (55, 56), resulting in underpowered studies. Measurements of different immune biomarkers made in the current study enable sample sizes for future studies in older people to be estimated. Using data for neutrophil and monocyte phagocytosis, sample sizes of between 10 and 30 per group would be required to identify a 20% increase in either percentage of active cells or MFI as significant. However, the current studies suggest that effect sizes for the probiotic combination used here may be much smaller than this, requiring sample sizes of several hundred to identify effects as significant. Using data for plasma markers of inflammation (e.g., concentrations of CRP, TNF-α, IL-6, sICAM-1, sVCAM-1 or sE-selectin), sample sizes of between 35 and 310 per group would be required to identify a 20% decrease in concentration as significant. Using data for LPS-stimulated production of cytokines (TNF-α, IL-1β, IL-6, IL-10), sample sizes of over 100 per group and perhaps as many as 400 per group would be required to identify a 20% change as significant. The antibody response to vaccination is considered to be a useful biomarker of immune function (51, 52), in part because it is a measure of an integrated response to an immunological challenge and in part because it avoids the confounding effects of *ex vivo* manipulations such as cell culture and of technical variations in those. However, the situation with respect to the response to influenza vaccine is complex because older people can have weak responses, the vaccine composition changes regularly (sometimes annually), and the response to the different viral strains within the vaccine is variable within an individual. The current study identified numerically better responses to all four stains of seasonal influenza vaccine in the probiotic group compared to the control group but only one of these (seroconversion to the A/Michigan/2015 strain) was significant. Boge et al. (37) studied the effect of *L. casei* DN-114 001 on the response to the seasonal influenza vaccine in older individuals in French care homes. In a pilot study involving 86 individuals in two groups, they identified numerically greater antibody responses, seroprotection and seroconversion to all three viral strains in the vaccine, but none of the differences observed was statistically significant. Effect sizes for the probiotic compared to placebo for antibody titres at 3 weeks post-vaccination were approximately 12.5% for the H1N1 and B strains and ~65% for the H3N2 strain (37). Effect sizes for seroprotection and seroconversion varied between ~12.5 and 55%. In a follow-up study in 222 individuals in two groups, once again the antibody response, seroprotection and seroconversion were numerically higher in the probiotic group than the control group. However, only antibody titres to the B strain, seroprotection to the H1N1 strain in those who were not seroprotected already, and seroconversion to the H3N2 and B strains were statistically significant. These observations indicate than a sample size of ~110 per group may not be sufficient to identify effects on all components of the antibody response to vaccination as significant, indicating that larger sample sizes are required. It is important to keep in mind that different species and strains of probiotics may have larger or smaller effects on the vaccination response than the organisms studied in the current trial and by Boge et al. (37) and this will influence the sample size necessary to identify an effect as significant. Furthermore, responses to other types of vaccination may produce different effect sizes.

In conclusion, intervention with the combination of LGG plus BB12 at a total dose of ~1.3 to 1.6 × 10^9^ CFU per day for at least 6 months did not have any effect on a broad range of immune biomarkers in older people resident in care homes, although there was an indication that the probiotics improved the response to seasonal influenza vaccination with significantly higher seroconversion to one strain of the quadrivalent vaccine. The findings of the study provide limited support at best for the use of these probiotics to improve the immune response in this population, although the small sample size means that any interpretation of the findings should be made with caution. The possible effects of these probiotics on the vaccination response need further exploration in a larger trial. Other probiotic organisms may be effective in improving the immune response.

## Data Availability Statement

The original contributions presented in the study are included in the article/Supplementary Material, further inquiries can be directed to the corresponding author/s.

## Ethics Statement

The studies involving human participants were reviewed and approved by Wales REC 3. The patients/participants provided their written informed consent to participate in this study.

## Author Contributions

MW, MLo, MLa, RL, KH, DG, FH, PL, CB, and PC conceptualized and designed the PRINCESS trial. EO-J and RL provided support for the PRINCESS trial. CB oversaw the conduct of the PRINCESS trial. VC-H and HF conducted laboratory research reported here (apart from the microbiology) under the supervision of EM and PC. MW supervised the microbiology. VC-H, KH, and DG conducted the statistical analysis. VC-H and PC drafted the manuscript. All authors commented on the manuscript and agreed the final version.

## Conflict of Interest

PC has acted as a consultant to Chr. Hansen, but not in the context of this trial. The remaining authors declare that the research was conducted in the absence of any commercial or financial relationships that could be construed as a potential conflict of interest.
