# Supplementary material for: Combination of the Probiotics Lacticaseibacillus rhamnosus GG and Bifidobacterium animalis subsp. lactis, BB-12 Has Limited Effect on Biomarkers of Immunity and Inflammation in Older People Resident in Care Homes: Results From the Probiotics to Reduce Infections iN CarE home reSidentS Randomized, Controlled Trial
Source: Front Immunol. 2021 Mar 4;12:643321. doi: 10.3389/fimmu.2021.643321 (PMC7969511; doi:10.3389/fimmu.2021.643321)
Supplement: Supplementary file 1 [file Table_1.DOCX]

Supplementary Material

# Supplementary Tables

Supplementary table 1. Components of the full blood count in participants in the placebo and probiotic groups

| Parameter and reference value (10^9^/L) | Placebo (n=30) |  | Probiotic (n=30) |  | Covariables | Adjusted mean difference  (95% CI) | | | p-value |
| --- | --- | --- | --- | --- | --- | --- | --- | --- | --- |
| Descriptive statistics categorised according to allocation | | | |  | ANCOVA Neutrophils _(LogTr)_ | | | | |
| Neutrophils (2.0 - 7.5) | Mean ± SE |  | Mean ± SE |  | Trial Arm | 0.006 | (-0.058 | 0.069) | 0.861 |
| Baseline | 4.8 ± 0.4 |  | 4.6 ± 0.4 |  | Sex | 0.017 | (-0.047 | 0.081) | 0.593 |
| Post-intervention | 4.4 ± 0.3 |  | 4.7 ± 0.3 |  | Baseline | 0.520 | (0.348 | 0.691) | < 0.001 |
| Descriptive statistics categorised according to allocation | | | |  | ANCOVA Lymphocytes _(LogTr)_ | | | | |
| Lymphocytes (1.5 - 5.0) | Mean ± SE |  | Mean ± SE |  | Trial Arm | 0.018 | (-0.047 | 0.082) | 0.583 |
| Baseline | 1.4 ± 0.1 |  | 2.4 ± 0.5 |  | Sex | 0.007 | (-0.055 | 0.068) | 0.826 |
| Post-intervention | 1.5 ± 0.1 |  | 2.5 ± 0.6 |  | Baseline | 0.796 | (0.651 | 0.940) | < 0.001 |
| Descriptive statistics categorised according to allocation | | | |  | ANCOVA Monocytes _(LogTr)_ | | | | |
| Monocytes (0.2 - 1.0) | Mean ± SE |  | Mean ± SE |  | Trial Arm | -0.023 | (-0.104 | 0.058) | 0.566 |
| Baseline | 0.5 ± 0.0 |  | 0.6 ± 0.0 |  | Sex | -0.020 | (-0.099 | 0.060) | 0.623 |
| Post-intervention | 0.5 ± 0.0 |  | 0.6 ± 0.1 |  | Baseline | 0.665 | (0.403 | 0.928) | < 0.001 |
| Descriptive statistics categorised according to allocation | | | |  | ANCOVA Total leukocytes _(LogTr)_ | | | | |
| Total leukocytes (4 – 11.0) | Mean ± SE |  | Mean ± SE |  | Trial Arm | 0.014 | (-0.035 | 0.064) | 0.563 |
| Baseline | 6.9 ± 0.4 |  | 8.0 ± 0.6 |  | Sex | 0.006 | (-0.044 | 0.055) | 0.824 |
| Post-intervention | 7.1 ± 0.4 |  | 8.2 ± 0.7 |  | Baseline | 0.702 | (0.535 | 0.868) | < 0.001 |
| Descriptive statistics categorised according to allocation | | | |  | ANCOVA Platelets _(LogTr)_ | | | | |
| Platelets (140 - 400) | Mean ± SE |  | Mean ± SE |  | Trial Arm | 0.019 | (-0.021 | 0.060) | 0.347 |
| Baseline | 282 ± 16 |  | 292 ± 23 |  | Sex | -0.008 | (-0.054 | 0.037) | 0.718 |
| Post-intervention | 267 ± 21 |  | 284 ± 15 |  | Baseline | 0.832 | (0.668 | 0.995) | < 0.001 |
| Descriptive statistics categorised according to allocation | | | |  | Mann-Whitney Test – Factor Allocation | | | | |
| Eosinophils (0.0 - 0.5) | Mean ± SE |  | Mean ± SE |  | Eosinophils | p-value | | | |
| Baseline | 0.2 ± 0.0 |  | 0.2 ± 0.0 |  | Post-intervention | 0.816 | | | |
| Post-intervention | 0.2 ± 0.0 |  | 0.3 ± 0.0 |  |  |  |  |  |  |
| Basophils (0.0 - 0.1) | Mean ± SE |  | Mean ± SE |  | Basophils | p-value | | | |
| Baseline | 0.0 ± 0.0 |  | 0.1 ± 0.0 |  | Post-intervention | 0.688 | | | |
| Post-intervention | 0.1 ± 0.0 |  | 0.1 ± 0.1 |  |  |  |  |  |  |

ANCOVA modelling used log transformed (LogTr) data to determine the effects of trial arm (allocation: probiotic or placebo), sex, and baseline value with the post-intervention variable considered as the dependent outcome. Significance levels were defined as p-values <0.05. Eosinophils and basophils received a different statistical treatment to determine significance due to the nature of the variable (values including zero); thus the Mann-Whitney test was used. Significant levels were defined as p-values <0.05.

Supplementary table 2. Blood immune cell phenotypes in participants in the placebo and probiotic groups

| Variable (cells/µl) | Placebo (n=30) | Probiotic (n=30) |  | Covariable | Adjusted mean difference  (95% CI) | | | p-value |
| --- | --- | --- | --- | --- | --- | --- | --- | --- |
| Descriptive statistics categorised according to allocation | | |  | ANCOVA T cells _(LogTr)_ | | | | |
| T cells | Mean ± SE | Mean ± SE |  | Trial Arm | 0.03 | (-0.02 | 0.08) | 0.26 |
| Baseline | 1298 ± 51 | 1338 ± 79 |  | Sex | 0.04 | (-0.01 | 0.10) | 0.12 |
| Post-intervention | 1388 ± 65 | 1539 ± 89 |  | Baseline | 0.65 | (0.41 | 0.90) | < 0.001 |
| Descriptive statistics categorised according to allocation | | |  | ANCOVA T helper _(LogTr)_ | | | | |
| T helper cells | Mean ± SE | Mean ± SE |  | Trial Arm | 0.01 | (-0.06 | 0.07) | 0.79 |
| Baseline | 886 ± 64 | 912 ± 82 |  | Sex | 0.05 | (-0.02 | 0.11) | 0.17 |
| Post-intervention | 986 ± 73 | 1015 ± 80 |  | Baseline | 0.79 | (0.66 | 0.93) | < 0.001 |
| Descriptive statistics categorised according to allocation | | |  | ANCOVA T regs _(LogTr)_ | | | | |
| T regs | Mean ± SE | Mean ± SE |  | Trial Arm | 0.02 | (-0.08 | 0.12) | 0.71 |
| Baseline | 87 ± 15 | 86 ± 14 |  | Sex | -0.05 | (-0.16 | 0.06) | 0.34 |
| Postintervention | 80 ± 14 | 84 ± 13 |  | Baseline | 0.79 | (0.67 | 0.92) | < 0.001 |
| Descriptive statistics categorised according to allocation | | |  | ANCOVA T cytotoxic _(LogTr)_ | | | | |
| T cytotoxic cells | Mean ± SE | Mean ± SE |  | Trial Arm | -0.01 | (-0.06 | 0.04) | 0.67 |
| Baseline | 631 ± 35 | 767 ± 33 |  | Sex | -0.04 | (-0.08 | 0.00) | 0.07 |
| Postintervention | 717 ± 39 | 803 ± 38 |  | Baseline | 0.74 | (0.58 | 0.90) | < 0.001 |
| Descriptive statistics categorised according to allocation | | |  | ANCOVA Activated T cytotoxic _(LogTr)_ | | | | |
| Activated T cytotoxic cells | Mean ± SE | Mean ± SE |  | Trial Arm | -0.02 | (-0.09 | 0.05) | 0.59 |
| Baseline | 231 ± 18 | 270 ± 19 |  | Sex | -0.02 | (-0.10 | 0.05) | 0.48 |
| Postintervention | 279 ± 14 | 282 ± 21 |  | Baseline | 0.10 | (-0.05 | 0.26) | 0.19 |
| Descriptive statistics categorised according to allocation | | |  | ANCOVA Ratio CD4^+^:CD8^+^ _(LogTr)_ | | | | |
| Ratio CD4^+^:CD8^+^ | Mean ± SE | Mean ± SE |  | Trial Arm | 0.02 | (-0.05 | 0.10) | 0.56 |
| Baseline | 1.4 ± 0.1 | 1.2 ± 0.1 |  | Sex | 0.09 | (0.02 | 0.16) | 0.02 |
| Postintervention | 1.4 ± 0.1 | 1.3 ± 0.1 |  | Baseline | 0.76 | (0.56 | 0.95) | < 0.001 |
| Descriptive statistics categorised according to allocation | | |  | ANCOVA NK cells _(LogTr)_ | | | | |
| NK cells | Mean ± SE | Mean ± SE |  | Trial Arm | 0.04 | (-0.04 | 0.12) | 0.33 |
| Baseline | 81 ± 5 | 82 ± 6 |  | Sex | 0.03 | (-0.05 | 0.11) | 0.46 |
| Postintervention | 73 ± 5 | 79 ± 4 |  | Baseline | 0.33 | (0.04 | 0.63) | 0.03 |
| Descriptive statistics categorised according to allocation | | |  | ANCOVA B cells _(LogTr)_ | | | | |
| B cells | Mean ± SE | Mean ± SE |  | Trial Arm | -0.04 | (-0.09 | 0.01) | 0.10 |
| Baseline | 221 ± 20 | 240 ± 20 |  | Sex | 0.02 | (-0.02 | 0.07) | 0.34 |
| Postintervention | 224 ± 21 | 232 ± 20 |  | Baseline | 0.84 | (0.73 | 0.95) | < 0.001 |
| Descriptive statistics categorised according to allocation | | |  | ANCOVA B cells CD80^+^ _(LogTr)_ | | | | |
| B cells CD80^+^ | Mean ± SE | Mean ± SE |  | Trial Arm | 0.00 | (-0.05 | 0.05) | 0.96 |
| Baseline | 138 ± 11 | 149 ± 16 |  | Sex | -0.01 | (-0.05 | 0.04) | 0.81 |
| Postintervention | 127 ± 12 | 143 ± 17 |  | Baseline | 0.86 | (0.74 | 0.98) | < 0.001 |
| Descriptive statistics categorised according to allocation | | |  | ANCOVA B cells CD86^+^ _(LogTr)_ | | | | |
| B cells CD86^+^ | Mean ± SE | Mean ± SE |  | Trial Arm | -0.02 | (-0.07 | 0.03) | 0.52 |
| Baseline | 142 ± 12 | 156 ± 17 |  | Sex | -0.03 | (-0.08 | 0.02) | 0.23 |
| Postintervention | 140 ± 14 | 152 ± 18 |  | Baseline | 0.88 | (0.76 | 1.01) | < 0.001 |
| Descriptive statistics categorised according to allocation | | |  | ANCOVA Monocytes _(LogTr)_ | | | | |
| Monocytes | Mean ± SE | Mean ± SE |  | Trial Arm | -0.02 | (-0.11 | 0.07) | 0.60 |
| Baseline | 448 ± 32 | 578 ± 38 |  | Sex | 0.00 | (-0.09 | 0.08) | 0.92 |
| Postintervention | 504 ± 41 | 570 ± 40 |  | Baseline | 0.80 | (0.57 | 1.02) | < 0.001 |
| Descriptive statistics categorised according to allocation | | |  | ANCOVA Monocytes CD80^+^ _(LogTr)_ | | | | |
| Monocytes CD80^+^ | Mean ± SE | Mean ± SE |  | Trial Arm | 0.02 | (-0.11 | 0.16) | 0.75 |
| Baseline | 155 ± 21 | 164 ± 26 |  | Sex | -0.07 | (-0.21 | 0.07) | 0.30 |
| Postintervention | 113 ± 18 | 120 ± 23 |  | Baseline | 0.71 | (0.52 | 0.91) | < 0.001 |
| Descriptive statistics categorised according to allocation | | |  | ANCOVA Monocytes CD86^+^ _(LogTr)_ | | | | |
| Monocytes CD86^+^ | Mean ± SE | Mean ± SE |  | Trial Arm | 0.07 | (-0.14 | 0.28) | 0.50 |
| Baseline | 131 ± 18 | 122 ± 20 |  | Sex | 0.04 | (-0.17 | 0.25) | 0.71 |
| Postintervention | 111 ± 20 | 124 ± 22 |  | Baseline | 0.87 | (0.65 | 1.08) | < 0.001 |

ANCOVA modelling used log transformed (LogTr) data to determine the effects of trial arm (allocation: probiotic or placebo), sex, and baseline value with the post-intervention variable considered as the dependent outcome. Significance levels were defined as p-values <0.05.

Supplementary table 3. Phagocyte function of neutrophils and monocytes from participants in the placebo and probiotic groups

| Variable | Placebo (n=30) | Probiotic (n=30) |  | Covariable | Adjusted mean difference  (95% CI) | | | p-value |
| --- | --- | --- | --- | --- | --- | --- | --- | --- |
| Descriptive statistics categorised according to Trial Arm | | |  | ANCOVA Phagocytic Activity Neutrophils % _(LogTr)_ | | | | |
| Phagocytic activity Neutrophils (%) | Mean ± SE | Mean ± SE |  | Trial Arm | 0.006 | (-0.028 | 0.039) | 0.727 |
| Baseline | 78.7 ± 1.8 | 81.2 ± 1.4 |  | Sex | 0.024 | (-0.010 | 0.058) | 0.158 |
| Post-intervention | 81.8 ± 2.1 | 83.1 ± 2.5 |  | Baseline | 0.207 | (0.028 | 0.386) | 0.025 |
| Descriptive statistics categorised according to Trial Arm | | |  | ANCOVA Phagocytic Activity Neutrophils GMFL _(LogTr)_ | | | | |
| Phagocytic activity Neutrophils (GMFL) | Mean ± SE | Mean ± SE |  | Trial Arm | 0.014 | (-0.052 | 0.081) | 0.666 |
| Baseline | 272.8 ± 10.2 | 243.4 ± 9.5 |  | Sex | 0.057 | (-0.008 | 0.123) | 0.084 |
| Post-intervention | 247.7 ± 11.4 | 245.2 ± 14.8 |  | Baseline | 0.398 | (0.145 | 0.650) | 0.003 |
| Descriptive statistics categorised according to Trial Arm | | |  | ANCOVA Phagocytic Activity Monocytes % _(LogTr)_ | | | | |
| Phagocytic activity Monocytes (%) | Mean ± SE | Mean ± SE |  | Trial Arm | 0.032 | (-0.069 | 0.134) | 0.524 |
| Baseline | 33.9 ± 1.6 | 28.3 ± 1.4 |  | Sex | 0.001 | (-0.098 | 0.100) | 0.979 |
| Post-intervention | 29.6 ± 1.9 | 29.5 ± 2.1 |  | Baseline | 0.333 | (0.100 | 0.566) | 0.006 |
| Descriptive statistics categorised according to Trial Arm | | |  | ANCOVA Phagocytic Activity Monocytes GMFL _(LogTr)_ | | | | |
| Phagocytic activity Monocytes (GMFL) | Mean ± SE | Mean ± SE |  | Trial Arm | 0.028 | (-0.048 | 0.104) | 0.465 |
| Baseline | 182.3 ± 8.7 | 184.3 ± 11.0 |  | Sex | -0.013 | (-0.089 | 0.063) | 0.738 |
| Post-intervention | 174.0 ± 9.5 | 184.9 ± 11.6 |  | Baseline | 0.039 | (-0.176 | 0.254) | 0.070 |

ANCOVA modelling used log transformed (LogTr) data to determine the effects of trial arm (allocation: probiotic or placebo), sex, and baseline value with the post-intervention variable considered as the dependent outcome. Significance levels were defined as p-values <0.05. GMFL=Geometric Mean Fluorescence intensity

Supplementary table 4. Plasma immune mediator concentrations in participants in the placebo and probiotic groups

| Variable | Placebo (n=18) | Probiotic (n=18) | |  | Covariable | | Adjusted mean difference  (95% CI) | | | p-value | |
| --- | --- | --- | --- | --- | --- | --- | --- | --- | --- | --- | --- |
| Descriptive statistics categorised according to trial arm | | | |  | ANCOVA ICAM-1 _(LogTr)_ | | | | | | |
| ICAM-1 (ng/ml) | Mean ± SE | | Mean ± SE |  | Trial arm | | 0.042 | (-0.032 | 0.117) | 0.252 | |
| Baseline | 460 ± 42 | | 425 ± 33 |  | Sex | | 0.066 | (-0.01 | 0.142) | 0.087 | |
| Post-intervention | 425 ± 64 | | 440 ± 53 |  | Baseline | | 0.916 | (0.740 | 1.092) | < 0.001 | |
| Descriptive statistics categorised according to trial arm | | | |  | ANCOVA IL-1ra _(LogTr)_ | | | | | | |
| IL-1ra (ng/ml) | Mean ± SE | | Mean ± SE |  | Trial arm | | 0.032 | (-0.148 | 0.212) | 0.718 | |
| Baseline | 2.24 ± 0.34 | | 2.23 ± 0.27 |  | Sex | | 0.120 | (-0.062 | 0.303) | 0.189 | |
| Post-intervention | 2.49 ± 0.68 | | 2.52 ± 0.44 |  | Baseline | | 0.761 | (0.496 | 1.026) | < 0.001 | |
| Descriptive statistics categorised according to trial arm | | | |  | ANCOVA E-Selectin _(LogTr)_ | | | | | | |
| E-Selectin (ng/ml) | Mean ± SE | | Mean ± SE |  | Trial arm | | 0.011 | (-0.108 | 0.129) | 0.853 | |
| Baseline | 25.0 ± 2.2 | | 25.1 ± 1.9 |  | Sex | | -0.032 | (-0.150 | 0.087) | 0.591 | |
| Post-intervention | 24.2 ± 2.7 | | 25.4 ± 3.8 |  | Baseline | | 0.757 | (0.523 | 0.991) | < 0.001 | |
| Descriptive statistics categorised according to trial arm | | | |  | ANCOVA VCAM-1 _(LogTr)_ | | | | | | |
| VCAM-1 (ng/ml) | Mean ± SE | | Mean ± SE |  | Trial arm | | -0.003 | (-0.120 | 0.115) | 0.964 | |
| Baseline | 1040 ± 75 | | 804 ± 53 |  | Sex | | 0.025 | (-0.092 | 0.141) | 0.671 | |
| Post- intervention | 1152 ± 199 | | 914 ± 108 |  | Baseline | | 1.013 | (0.693 | 1.334) | < 0.001 | |
| Descriptive statistics categorised according to trial arm | | | |  | ANCOVA MCP-1 _(LogTr)_ | | | | | | |
| MCP-1 (ng/ml) | Mean ± SE | | Mean ± SE |  | Trial arm | | 0.073 | (-0.047 | 0.193) | 0.224 | |
| Baseline | 0.4 ± 0.04 | | 0.4 ± 0.03 |  | Sex | | 0.049 | (-0.074 | 0.172) | 0.423 | |
| Post-intervention | 0.4 ± 0.05 | | 0.4 ± 0.04 |  | Baseline | | 0.670 | (0.348 | 0.991) | < 0.001 | |
| Descriptive statistics categorised according to trial arm | | | |  | ANCOVA IP-10 _(LogTr)_ | | | | | | |
| IP-10 (ng/ml) | Mean ± SE | | Mean ± SE |  | Trial arm | | -0.002 | (-0.162 | 0.158) | 0.981 | |
| Baseline | 0.2 ± 0.01 | | 0.2 ± 0.01 |  | Sex | | -0.008 | (-0.176 | 0.16) | 0.923 | |
| Post-intervention | 0.1 ± 0.02 | | 0.1 ± 0.01 |  | Baseline | | 0.409 | (-0.016 | 0.834) | 0.059 | |
| Descriptive statistics categorised according to trial arm | | | |  | ANCOVA IL-17A _(LogTr)_ | | | | | | |
| IL-17A (ng/ml) | Mean ± SE | | Mean ± SE |  | Trial arm | | 0.076 | (-0.191 | 0.342) | 0.567 | |
| Baseline | 2.3 ± 0.5 | | 2.5 ± 0.4 |  | Sex | | -0.183 | (-0.445 | 0.08) | 0.166 | |
| Post-intervention | 3.0 ± 0.8 | | 3.1 ± 0.7 |  | Baseline | | 0.353 | (0.041 | 0.664) | 0.028 | |
| Descriptive statistics categorised according to trial arm | | | |  | ANCOVA TNFR-II _(LogTr)_ | | | | | | |
| TNFR-II (ng/ml) | Mean ± SE | | Mean ± SE |  | Trial arm | | 0.016 | (-0.075 | 0.106) | 0.728 | |
| Baseline | 4.0 ± 0.3 | | 5.0 ± 0.4 |  | Sex | | 0.070 | (-0.019 | 0.159) | 0.120 | |
| Post-intervention | 3.3 ± 0.4 | | 4.0 ± 0.5 |  | Baseline | | 0.685 | (0.473 | 0.898) | < 0.001 | |
| Descriptive statistics categorised according to trial arm | | | |  | ANCOVA IL-6 _(LogTr)_ | | | | | | |
| IL-6 (pg/ml) | Mean ± SE | | Mean ± SE |  | Trial arm | | -0.006 | (-0.299 | 0.287) | 0.966 | |
| Baseline | 7.9 ± 1.5 | | 12.5 ± 4.2 |  | Sex | | 0.049 | (-0.246 | 0.344) | 0.738 | |
| Post-intervention | 9.8 ± 3.4 | | 6.6 ± 1.6 |  | Baseline | | 0.199 | (-0.221 | 0.62) | 0.340 | |
| Descriptive statistics categorised according to trial arm | | | |  | ANCOVA IL-10 _(LogTr)_ | | | | | | |
| IL-10 (pg/ml) | Mean ± SE | | Mean ± SE |  | Trial arm | | -0.098 | (-0.369 | 0.172) | 0.463 | |
| Baseline | 0.9 ± 0.2 | | 0.9 ± 0.1 |  | Sex | | 0.342 | (0.065 | 0.62) | 0.017 | |
| Post-intervention | 0.7 ± 0.1 | | 0.7 ± 0.2 |  | Baseline | | 0.639 | (0.361 | 0.917) | < 0.001 | |
| Descriptive statistics categorised according to trial arm | | | |  | ANCOVA IL-12p70 _(LogTr)_ | | | | | | |
| IL-12p70 (pg/ml) | Mean ± SE | | Mean ± SE |  | Trial arm | | 0.154 | (-0.285 | 0.593) | 0.448 | |
| Baseline | 1.5 ± 0.3 | | 1.6 ± 0.2 |  | Sex | | -0.087 | (-0.554 | 0.381) | 0.685 | |
| Post-intervention | 1.2 ± 0.4 | | 1.6 ± 0.4 |  | Baseline | | 0.268 | (-0.607 | 1.143) | 0.506 | |
| Descriptive statistics categorised according to trial arm | | | |  | ANCOVA TNF-α _(LogTr)_ | | | | | | |
| TNF-α (pg/ml) | Mean ± SE | | Mean ± SE |  | Trial arm | | 0 | (-0.076 | 0.077) | 0.991 | |
| Baseline | 17.0 ± 1.1 | | 19.0 ± 1.6 |  | Sex | | 0.052 | (-0.03 | 0.135) | 0.205 | |
| Post-intervention | 17.1 ± 1.2 | | 17.4 ± 1.2 |  | Baseline | | 0.626 | (0.325 | 0.927) | < 0.001 | |
| Descriptive statistics categorised according to trial arm | | | | | | ANCOVA CRP _(LogTr)_ | | | | |  |
| CRP (mg/l) | Mean ± SE | | Mean ± SE |  | Trial Arm | | 1.66 | (-0.798 | 4.118) | 0.182 | |
| Baseline | 6.32 ± 2.16 | | 6.26 ± 1.37 |  | Sex | | -1.87 | (-4.357 | 0.618) | 0.136 | |
| Post-intervention | 4.62 ± 0.79 | | 6.10 ± 1.33 |  | Baseline | | 0.454 | (0.322 | 0.585) | < 0.001 | |

ANCOVA modelling used log transformed (LogTr) data to determine the effects of trial arm (allocation: probiotic or placebo), sex, and baseline value with the post-intervention variable considered as the dependent outcome. Significance levels were defined as p-values <0.05.

Supplementary table 5. *Ex vivo* responses of LPS-stimulated cultures of whole blood from participants in the placebo and probiotic groups

| Variable | Placebo (n=18) | | | Probiotic (n=18) | Covariable | Adjusted mean difference  (95% CI) | | | p-value |
| --- | --- | --- | --- | --- | --- | --- | --- | --- | --- |
| Descriptive statistics categorised according to Trial Arm | | | | | ANCOVA IL-10 LPS stimulated _(LogTr)_ | | | | |
| IL-10 (ng/ml) |  | Mean ± SE |  | Mean ± SE | Trial Arm | -0.044 | (-0.301 | 0.213) | 0.730 |
| Baseline |  | 4.3 ± 1.7 |  | 4.2 ± 1.4 | Sex | -0.127 | (-0.388 | 0.134) | 0.328 |
| Post-intervention |  | 3.7 ± 0.7 |  | 3.9 ± 0.8 | Baseline | 0.376 | (0.128 | 0.625) | 0.004 |
| Descriptive statistics categorised according to Trial Arm | | | | | ANCOVA TNF-α LPS stimulated _(LogTr)_ | | | | |
| TNF-α (ng/ml) |  | Mean ± SE |  | Mean ± SE | Trial Arm | -0.051 | (-0.28 | 0.179) | 0.655 |
| Baseline |  | 10.9 ± 2.3 |  | 13.7 ± 2.6 | Sex | -0.099 | (-0.326 | 0.129) | 0.384 |
| Post-intervention |  | 11.4 ± 1.9 |  | 12.1 ± 2.4 | Baseline | 0.249 | (-0.053 | 0.551) | 0.103 |
| Descriptive statistics categorised according to Trial Arm | | | | | ANCOVA IL-6 LPS stimulated _(LogTr)_ | | | | |
| IL-6 (ng/ml) |  | Mean ± SE |  | Mean ± SE | Trial Arm | -0.037 | (-0.209 | 0.135) | 0.664 |
| Baseline |  | 40.2 ± 5.3 |  | 47.6 ± 6.9 | Sex | -0.081 | (-0.256 | 0.094) | 0.351 |
| Post-intervention |  | 43.8 ± 4.6 |  | 45.7 ± 6.5 | Baseline | 0.261 | (-0.036 | 0.557) | 0.083 |
| Descriptive statistics categorised according to Trial Arm | | | | | ANCOVA IL-1β LPS stimulated _(LogTr)_ | | | | |
| IL-1β (ng/ml) |  | Mean ± SE |  | Mean± SE | Trial Arm | 0.095 | (-0.205 | 0.395) | 0.522 |
| Baseline |  | 3.8 ± 0.8 |  | 6.4 ± 2.8 | Sex | -0.274 | (-0.576 | 0.029) | 0.075 |
| Post-intervention |  | 4.3 ± 0.7 |  | 15.1 ± 8.8 | Baseline | 0.171 | (-0.238 | 0.581) | 0.399 |

ANCOVA modelling used log transformed (LogTr) data to determine the effects of trial arm (allocation: probiotic or placebo), sex, and baseline value with the post-intervention variable considered as the dependent outcome. Significance levels were defined as p-values <0.05.

Supplementary table 6. *Ex vivo* responses of PGN-stimulated cultures of whole blood from participants in the placebo and probiotic groups

| Variable | Placebo (n=18) | Probiotic (n=18) | Covariable | Adjusted mean difference  (95% CI) | | | p-value |
| --- | --- | --- | --- | --- | --- | --- | --- |
| Descriptive statistics categorised according to Trial Arm | | | ANCOVA IL-10 PGN stimulated _(LogTr)_ | | | | |
| IL-10 (pg/ml) | Mean ± SE | Mean± SE | Trial Arm | -0.103 | (-0.349 | 0.142) | 0.396 |
| Baseline | 346 ± 58 | 583 ± 133 | Sex | -0.272 | (-0.524 | -0.02) | 0.035 |
| Post-intervention | 580 ± 93 | 504 ± 99 | Baseline | -0.354 | (-0.614 | -0.094) | 0.009 |
| Descriptive statistics categorised according to Trial Arm | | | ANCOVA TNF-α PGN stimulated _(LogTr)_ | | | | |
| TNF-α (pg/ml) | Mean ± SE | Mean ± SE | Trial Arm | -0.123 | (-0.407 | 0.162) | 0.386 |
| Baseline | 2377 ± 726 | 3766 ± 1003 | Sex | -0.114 | (-0.393 | 0.166) | 0.413 |
| Post-intervention | 2675 ± 495 | 2130 ± 407 | Baseline | -0.061 | (-0.352 | 0.23) | 0.672 |
| Descriptive statistics categorised according to Trial Arm | | | ANCOVA IL-6 PGN stimulated _(LogTr)_ | | | | |
| IL-6 (ng/ml) | Mean ± SE | Mean ± SE | Trial Arm | -0.12 | (-0.401 | 0.16) | 0.388 |
| Baseline | 36.2 ± 7.2 | 70.9 ± 17.7 | Sex | -0.274 | (-0.556 | 0.009) | 0.057 |
| Post-intervention | 54.9 ± 7.3 | 54.0 ± 10.3 | Baseline | -0.148 | (-0.457 | 0.162) | 0.339 |
| Descriptive statistics categorised according to Trial Arm | | | ANCOVA IL-1β PGN stimulated _(LogTr)_ | | | | |
| IL-1β (pg/ml) | Mean ± SE | Mean ± SE | Trial | -0.244 | (-0.639 | 0.15) | 0.216 |
| Baseline | 166 ± 45.1 | 321 ± 123 | Sex | -0.137 | (-0.54 | 0.266) | 0.492 |
| Post-intervention | 340 ± 93.8 | 214 ± 61 | Baseline | -0.049 | (-0.316 | 0.218) | 0.711 |

ANCOVA modelling used log transformed (LogTr) data to determine the effects of trial arm (allocation: probiotic or placebo), sex, and baseline value with the post-intervention variable considered as the dependent outcome. Significance levels were defined as p-values <0.05.

Supplementary table 7. *Ex vivo* responses of PHA-stimulated cultures of whole blood from participants in the placebo and probiotic groups

| Variable | Placebo (n=18) | Probiotic (n=18) | Covariable | Adjusted mean difference  (95% CI) | | | p-value |
| --- | --- | --- | --- | --- | --- | --- | --- |
| Descriptive statistics categorised according to Trial Arm | | | ANCOVA IFN-γ PHA stimulated _(LogTr)_ | | | | |
| IFN-γ (pg/ml) | Mean ± SE | Mean ± SE | Trial Arm | 0.045 | -0.777 | 0.867 | 0.912 |
| Baseline | 23.2 ± 17.1 | 21.3 ± 8.8 | Sex | 0.079 | -0.713 | 0.870 | 0.841 |
| Post-intervention | 18.5 ± 9.4 | 16.5 ± 5.6 | Baseline | 0.383 | -0.019 | 0.785 | 0.061 |
| Descriptive statistics categorised according to Trial Arm | | | ANCOVA TNF-α PHA stimulated _(LogTr)_ | | | | |
| TNF-α (pg/ml) | Mean ± SE | Mean ± SE | Trial Arm | -0.024 | -0.197 | 0.148 | 0.776 |
| Baseline | 1343 ± 156 | 2013 ± 168 | Sex | 0.015 | -0.150 | 0.179 | 0.854 |
| Post-intervention | 1804 ± 224 | 2095 ± 192 | Baseline | 0.298 | -0.015 | 0.610 | 0.061 |

ANCOVA modelling used log transformed (LogTr) data to determine the effects of trial arm (allocation: probiotic or placebo), sex, and baseline value with the post-intervention variable considered as the dependent outcome. Significance levels were defined as p-values <0.05.
